# Myeloid-derived suppressor cells therapy enhance immunoregulatory properties in acute graft versus host disease with combination of regulatory T cells

**DOI:** 10.1186/s12967-020-02657-6

**Published:** 2020-12-14

**Authors:** Min-Jung Park, Jin-Ah Baek, Se-Young Kim, Kyung-Ah Jung, Jeong Won Choi, Sung-Hwan Park, Seung‐Ki Kwok, Mi-La Cho

**Affiliations:** 1 The Rheumatism Research Center, Catholic Research Institute of Medical Science, The Catholic University of Korea, 505 Banpo-dong, Seocho-gu, Seoul, 137-040 South Korea; 2 Divison of Rheumatology, Department of Internal Medicine, Seoul St. Mary’s Hospital, College of Medicine, The Catholic University of Korea, Seoul, South Korea

**Keywords:** Myeloid-derived suppressor cells, Regulatory T cells, Graft-versus-host disease, Immune tolerance, Cell therapy

## Abstract

**Background:**

Myeloid-derived suppressor cells (MDSCs) play a critical role in modulating the immune response and promoting immune tolerance in models of autoimmunity and transplantation. Regulatory T cells (Tregs) exert therapeutic potential due to their immunomodulatory properties, which have been demonstrated both in vitro and in clinical trials. Cell-based therapy for acute graft-versus-host disease (aGVHD) may enable induction of donor-specific tolerance in the preclinical setting.

**Methods:**

We investigated whether the immunoregulatory activity of the combination of MDSCs and Tregs on T cell and B cell subset and alloreactive T cell response. We evaluated the therapeutic effects of combined cell therapy for a murine aGVHD model following MHC-mismatched bone marrow transplantation. We compared histologic analysis from the target tissues of each groups were and immune cell population by flow cytometric analysis.

**Results:**

We report a novel approach to inducing immune tolerance using a combination of donor-derived MDSCs and Tregs. The combined cell-therapy modulated in vitro the proliferation of alloreactive T cells and the Treg/Th17 balance in mice and human system. Systemic infusion of MDSCs and Tregs ameliorated serverity and inflammation of aGVHD mouse model by reducing the populations of proinflammatory Th1/Th17 cells and the expression of proinflammatory cytokines in target tissue. The combined therapy promoted the differentiation of allogeneic T cells toward Foxp3 + Tregs and IL-10-producing regulatory B cells. The combination treatment control also activated human T and B cell subset.

**Conclusions:**

Therefore, the combination of MDSCs and Tregs has immunomodulatory activity and induces immune tolerance to prevent of aGVHD severity. This could lead to the development of new clinical approaches to the prevent aGVHD.

## Background

Graft-versus-host disease (GVHD) is the major complications after allogeneic hematopoietic stem cell transplantation (Allo-HSCT) [1, 2]. During GVHD, allogenic T cells are differentiated into effector lineages and secrete proinflammatory cytokines [3]. Allogeneic T-cell response are suppressed by immunosuppressive drugs, reducing the risk for acute GVHD [4, 5]. However, immunosuppressive drug for GVHD result in toxic side-effects. Thus, there is an unmet need for novel treatment strategies for GVHD with less toxicity and fewer side effects.

Myeloid-derived suppressor cells (MDSCs) are a heterogeneous population of immature myeloid cells that negatively regulate the immune response [6, 7]. MDSC-mediated immunosuppression involves increased arginase-1 and inducible nitric oxide synthetase (iNOS) activity and induction of T-cell apoptosis. MDSCs are effective against experimental autoimmune rheumatoid arthritis, systemic lupus erythematosus (SLE), and inflammatory bowel disease (IBD) [8–10]. Recently, MDSCs also promote immune tolerance in the context of organ transplantation [11, 12], and have therapeutic potential in T-cell-mediated diseases, but their efficacy is controversial.

MDSCs can be differentiated and expanded in vitro by various methods using a variety of combinations of progenitor cells and cytokines [13]. MDSCs generated in vitro suppress autoimmune lupus [14] and prevent in a pre-clinical GVHD model [15–17], indicating therapeutic potential for T-cell-mediated diseases.

Regulatory T (Tregs) cells exert an immunoregulatory role and induce Immunological tolerance [18, 19]. CD4 + CD25 + Foxp3 + Treg-based cellular therapy is effective for recipients of bone marrow and solid organ transplantation [20–22] and in patients with autoimmune diseases [23]. Donor-derived Tregs are typically used, as they share an MHC type with CD4 + and CD8 + T cells, which are primarily responsible for GVHD [24]. However, the therapeutic potential of Tregs is limited by their short lifespan and their plasticity under pathological conditions [25–27].

MDSC and Treg cell interactions involve a positive feedback signals in which MDSCs expand Treg cells while Treg cells control MDSC function [28]. MDSC support the induction of regulatory B (Breg) cells [14], type of B cell that releases IL-10 and and has immunosuppressive effects [29, 30]. Given this background, combined cell therapy using MDSCs and Treg cells may be beneficial for the treatment of aGVHD. We investigated the effects of the combination of MDSCs and Tregs on the induction of tolerance to MHC-mismatched transplants. The combination of MDSCs and Tregs reciprocally modulated the populations of alloreactive Th1/Th17 cells and Foxp3 + Treg cells. Furthermore, the combination therapy alleviated aGVHD clinically and histopathologically by regulating the effector T/B cell and Treg/Breg balance. These findings indicate that the combination of MDSCs and Tregs shows promise for alleviating aGVHD.

## Methods

### Mice

8-weeks-old C57BL/6 (B6, H-2b) and BALB/c (H-2d) female mice were purchased from Orient Bio (Sungnam, South Korea). The mice were maintained under specific-pathogen-free conditions in an animal facility with controlled humidity (55 ± 5%), 12/12 h light/dark cycle, and temperature (22 ± 1 °C). Mice were fed mouse chow and tap water ad libitum. All animal experiments were performed in accordance with the animal care and use committee of The Catholic University of Korea approved the protocols.

### Generation of MDSCs

Bone marrow mononuclear cells cells (BMMCs) were flushed out the bone marrow cavity of tibias and femurs with α-minimum essential medium (Invitrogen). BMMCs were cultured in 6 well plate at 1 × 10^6^ cells /mL in complete medium supplemented with GM‐CSF (20 ng/mL) and M‐CSF (20 ng/mL) (both from R&D Systems). After three days, they were harvested and stained with CD11c, CD11b, and Gr-1 antibodies after blocking Fc receptors (all from BD Biosciences). CD11c–, CD11b + , and Gr-1 + MDSC populations were sorted using a MoFlo cell sorter (Beckman Coulter). Human PBMC were cultured in 6 well plate at 1 × 10^6^ cells/ml in complete medium supplemented with GM‐CSF(20 ng/ml) and IL-6 (20 ng/ml) (both from R&D Systems). After three days, Lineage − /HLA-DR − /CD33 + /CD11b + human MDSC subsets were sorted using a MoFlo cell sorter (Beckman Coulter). The purity of the sorted MDSCs was > 95%.

### Generation of Tregs

Mouse splenic CD4 + T cell were isolated from spleen by using mouse anti-CD4 microbeads (Miltenyi Biotec, Germany). To isolate Treg cells, CD4 + T-cells were cultured with plate-bound anti-mouse CD3 (1 μg/mL; BD Biosciences), soluble anti-mous CD28 (1 μg/mL; BD Biosciences), anti-IFN-γ (2 μg/ml), anti-IL-4 (2 μg/ml), human recombinant transforming growth factor-β (TGF-β; 5 ng/mL; PeproTech, London, UK), and Retinal (0.1 μM; Sigma-Aldrich, St. Louis, MO)(16).

Human CD4 + T cells were isolated from PBMC by using human anti-CD4 microbeads (Miltenyi Biotec, Germany). To isolate human Treg cells, human CD4 + T-cells were cultured with plate-bound anti-human CD3 (1 μg/mL), soluble anti-human CD28 (1 μg/mL), anti-IFN-γ (2 μg/ml), anti-IL-4 (2 μg/ml), human recombinant TGF-β (5 ng/mL) and Retinal (0.1 μM).

After three days, the induced Treg cells were stained with CD4, CD25 and then sorted using a MoFlo cell sorter to obtain a ~ 95% pure CD4 + CD25 + population.

### Alloreactive T-cell responses in vitro

Responder CD4 + T cells (responder cells) were derived from Balb/c mice. Antigen-presenting cells (APCs: T-cell-depleted splenocytes) were isolated from Balb/c (syngenic) or B6 (allogenic) mice and irradiated with 3,000 cGy. Responder cells (1 × 10^5^) and irradiated APCs(1 × 10^5^) were cocultured in 96-well plates for 4 days, pulsed with 1 μCi tritiated thymidine (3[H]-TdR; NEN Life Science Products Inc., Boston, MA) at 18 h before the end of the experiment, and counted using an automated harvester (PHD Cell Harvester; Cambridge Technology, Inc., Cambridge, MA).

### In vitro co-culture systems

In FACS analysis, mouse or human CD4 + T cells (1 × 10^6^) were cocultured with MDSC or Treg alone or combined MDSC (2 × 10^5^) and Treg (2 × 10^5^) cells for 3 days in the presence of anti-mouse CD3 antibody (0.5ug/ml). In the mixed lymphocyte reaction assay, responder CD4 + T cells (1 × 10^5^) were cocultured with MDSC or Treg alone or combined MDSC (2 × 10^4^) and Treg (2 × 10^4^) cells for 4 days in the presence of anti-mouse CD3 antibody (0.5ug/ml). For all experimental conditions, MDSCs or Treg to T cells ratio is 1:5.

### Bone marrow transplantation

To develop the aGVHD model, Balb/c were lethally irradiated with 700 cGy and infused with 5 × 10^6^ donor BM cells plus 5 × 10^6^ splenocytes from Balb/c(syngenic) or C57BL/6(donor, allogenic) on day 0. On day1 and day7 after bone marrow transplantation (BMT), recipient mice received MDSCs (1 × 10^6^) and Tregs (1 × 10^6^) individually or in combination. The mice were monitored for clinical signs and body weight. The clinical GVHD was scored twice weekly using the clinical GVHD scoring system (Additional file 1: Table S1) [31]. Each of the five clinical parameters summed up to get the final score at indicated time points.

### Histological and immunohistochemical analyses

Mice were sacrificed on day 28 after BMT and organs captured, cryoembedded, and sectioned. Tissue specimens were fixed in 10% formalin buffer and embedded in paraffin. Sections (6 μm thick) were stained with H&E and the histologic score was determined using established scoring systems [31, 32]. For immunohistochemistry staining, sections were stained with primary antibodies against IL-6 [Abcam, Cambridge, England (ab7737)], IL-17 [Abcam (ab79056)], TNF-α [Abcam (ab6671)] and Foxp3 [H-190, Santa Cruz Biotechnology (sc-28705)] overnight at 4 °C, followed by addition of a biotinylated secondary antibody and a streptavidin-peroxidase mixture for 1 h. Color was developed by addition of 3,3-diaminobenzidine (Dako, Carpinteria, CA).

### Flow cytometry analysis

Mouse lymphocytes were stained with following fluorochrome conjugated antibodies: CD4(L3T4)-PerCP Cy5.5, CD25(PC61)-APC, Foxp3(FJK-16 s)-PE, IFN-γ(XMG1.2)-APC, IL-17(eBio17B7)-FITC, B220(RA3-6B2)-APC, CD19(eBio1D3(1D3))-PerCP, CD1d(1B1)- PE, CD5(53–7.3)-FITC, IL-10(JES5-16E3)-APC, CD138(281–2)-PE, and T- and B-Cell Activation Antigen (GL7)-FITC. Human lymphocytes were stained with following fluorochrome conjugated antibodies: CD4(RPA-T4)-PECy7, CD25(BC96)-APC, Foxp3(259D/C7)-PE, IFN-γ(4S.B3)-APC, IL-17(eBio64DEC1-FITC, CD19(HIB19)-FITC, IL-10(JES3-19F1)-APC, CD138(MI15)-PB450. Before intracellular staining, cells were stimulated for 4 h with phorbol myristate acetate (25 ng/mL) and ionomycin (250 ng/mL) in the presence of Golgistop (BD Biosciences, San Jose, CA, USA). Intracellular staining was fixed using a BD Cytofix/ Cytoperm Plus Fixation/Permeabilization Kit and BD Golgistop Kit (BD Biosciences, San Jose, CA). Foxp3, transcription factor was fixed using a Foxp3/Transcription Factor Staining buffer set (eBioscience, San Diego, CA) following the manufacturer’s instructions. Flow cytometric analysis was performed using a cytoFLEX Flow Cytometer (Beckman Coulter, Brea, CA, USA) and collected data were analyzed using the FlowJo software (Tree Star, Ashland, OR).

### Enzyme-linked immunosorbent assay

The concentrations of IL-17 and IFN-γ in culture supernatants were measured in duplicate using a sandwich enzyme-linked immunosorbent assay (ELISA) according to the manufacturer’s protocol (DuoSet; R&D Systems, Lille, France).

### Statistical analyses

Data are expressed as the means ± standard error of the mean (SEM). One-way analysis of variance followed by Bonferroni’s post hoc test was used to compare the differences between three or more groups. Statistical significance was considered as *P* value < 0.05. All statistical analysis was performed with Prism (standard version 5.01; GraphPad Software, San Diego, CA).

## Results

### The combination of MDSCs and Tregs controls the T cell and B cell response

**Fig. 1:**
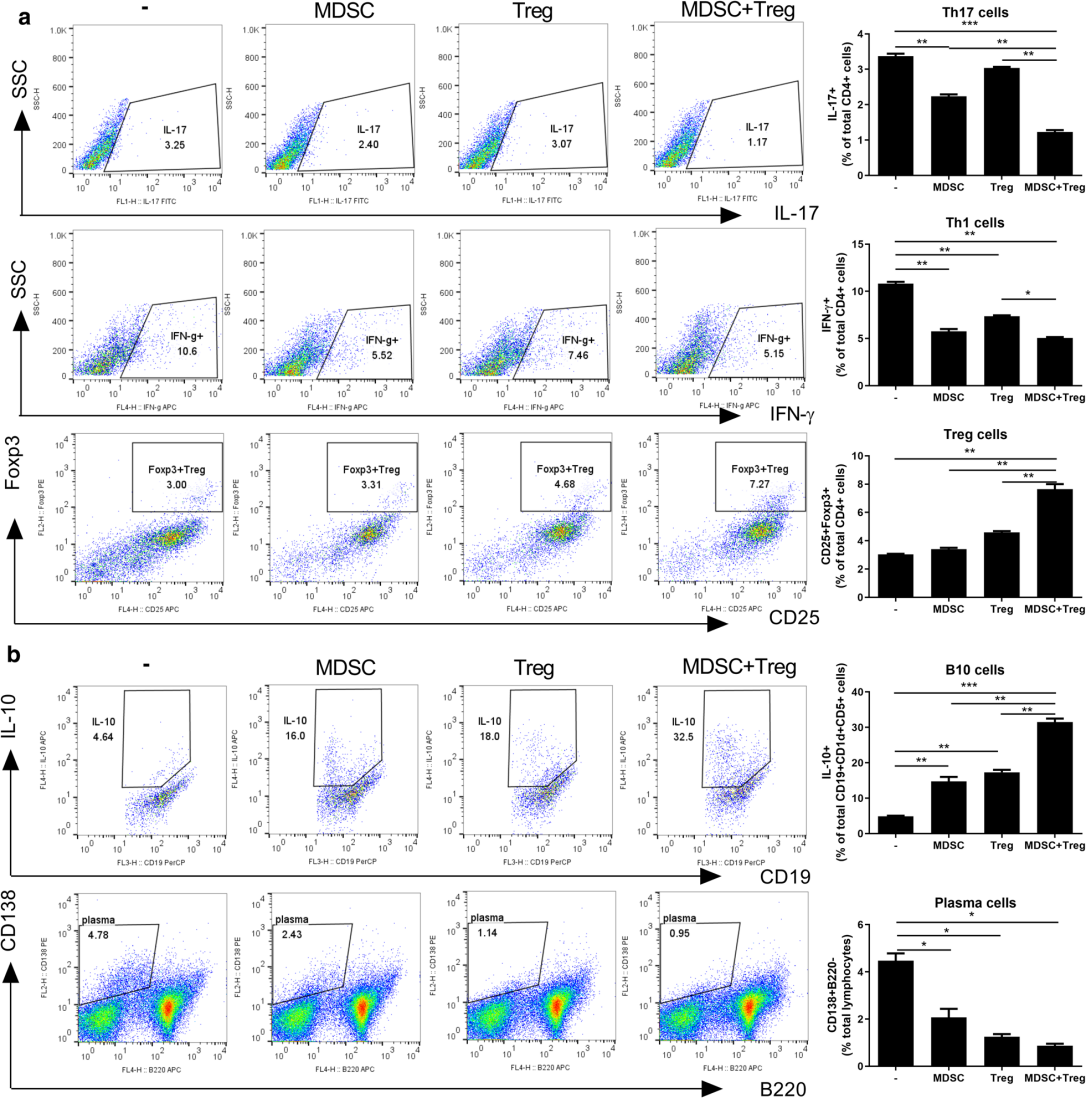
Combined treatment with MDSCs and Treg regulates T cell and B cell response.** a** CD4 + T cell(1 × 10^6^) isolated from normal C57BL/6 mice were cocultured with MDSC or Treg alone or combined MDSC(2 × 10^5^) and Treg (2 × 10^5^) cells for 3 days in the presence of anti-mouse CD3 antibody and analyzed by flow cytometry. A plot from one representative experiment displays the proportions of IL-17 + , IFN-γ + , CD25 + Foxp3 + cells among CD4 + T cells. Numbers in the plots indicate percentages of gated cells. **b** Total splenocytes (1 × 10^6^) of normal C57BL/6 mice coculture with MDSC or Treg alone or combined MDSC and Treg cells for 3 days in the presence of LPS (100 ng/ml) and analyzed by flow cytometry. A plot from one representative experiment displays the proportions of IL-10 + CD19 + cells, CD138 + B220- cells. Numbers in the plots indicate percentages of gated cells. Data are means ± SEMs. Data are representative of three independent experiments (*p < 0.05, **p < 0.01, ***p < 0.001)

To titrate of MDSC or Treg: T cells ratio in vitro coculture experiments, we performed using 3 dose (1/20:1,1/5:1,1:1 ratio, E:T ratio) evaluating Treg and Th17 cell regulation. However, MDSC or Treg: T cells (1:1) ratio is excluded in combined treatment. We chosen highly efficient ratio of 1:5 ratio in regulation of Treg/Th17 by Tregs and MDSCs combined therapy (Additional file 2: Figure S1). To evaluate the effects of the combination of MDSCs and Tregs on T- and B-cell subsets, we cultured CD4 + T cells from C57BL/6 spleen with MDSCs and Tregs individually or in combination for 3 days in vitro. The Th1 and Th17 cell populations were significantly reduced, and the Foxp3 + Treg population was significantly increased, by the combination treatment compared to either cell type alone (Fig. 1a). C57BL/6 splenocytes were incubated with LPS for 3 days in the presence of MDSCs and Tregs individually or in combination. The combination treatment markedly increased the population of IL-10-producing Bregs (Fig. 1b) and significantly reduced the plasma cell population compared to the control and MDSCS or Tregs alone (Fig. 1b).

**Fig. 2:**
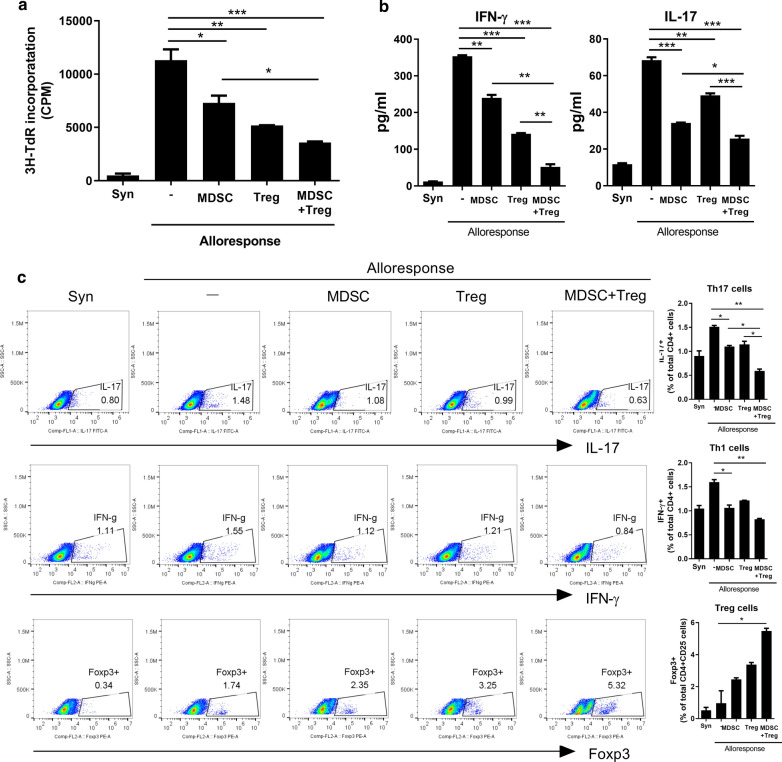
Combined treatment with MDSCs and Treg control alloreactive T cell response.** a** In the mixed lymphocyte reaction assay, a total of 1 × 10^5^ B/c splenic T cells (responders) were incubated with 1 × 10^5^ irradiated B/c (syngeneic stimulators, Syn) or B6 (allogeneic stimulators, Allo]) splenic APCs for 4 days. Responder cells were cultured in the presence or absence of MDSC (2 × 10^4^) or/and Treg (2 × 10^4^). **b** IFN-γ and IL-17 levels in the supernatants were measured by ELISA. **c** Intracellular immunostaining of IL-17 + CD4 + , IFN-γ + CD4 + , Foxp3 + Treg cells was performed were determined by flow cytometry. Data are means ± SEMs. Data are representative of three independent experiments (*p < 0.05, **p < 0.01, ***p < 0.001 versus alloresponse)

To determine whether combination treatement of MDSC and Treg has a inhibitory effect on proliferation of alloreactive T cells, we performed in vitro alloreactive proliferation assay. Alloreactive CD4^+^ T cells had proliferated vigorously to allogenic stimulation. MDSCs or Tregs alone suppressed the proliferation of alloreactive T cells. The combination more potently diminished the proliferation of alloreactive T cells (Fig. 2a). Under alloreactive T cell-activation conditions, elevated interferon (IFN)-γ and IL-17 levels were markedly reduced by the combination treatment, compared to treatment with either cell type alone (Fig. 2b).

Next, we assessed the effects of combined therapy with MDSCs and Tregs on alloreactive T cell subsets. Combined therapy increased the population of CD4^+^Foxp^+^ Treg cells approximately threefold (Fig. 2c) but reduced the population of effector Th1 and Th17 cells (Fig. 2c).

### The combination of MDSCs and Tregs ameliorates acute GVHD severity and inflammation

**Fig. 3:**
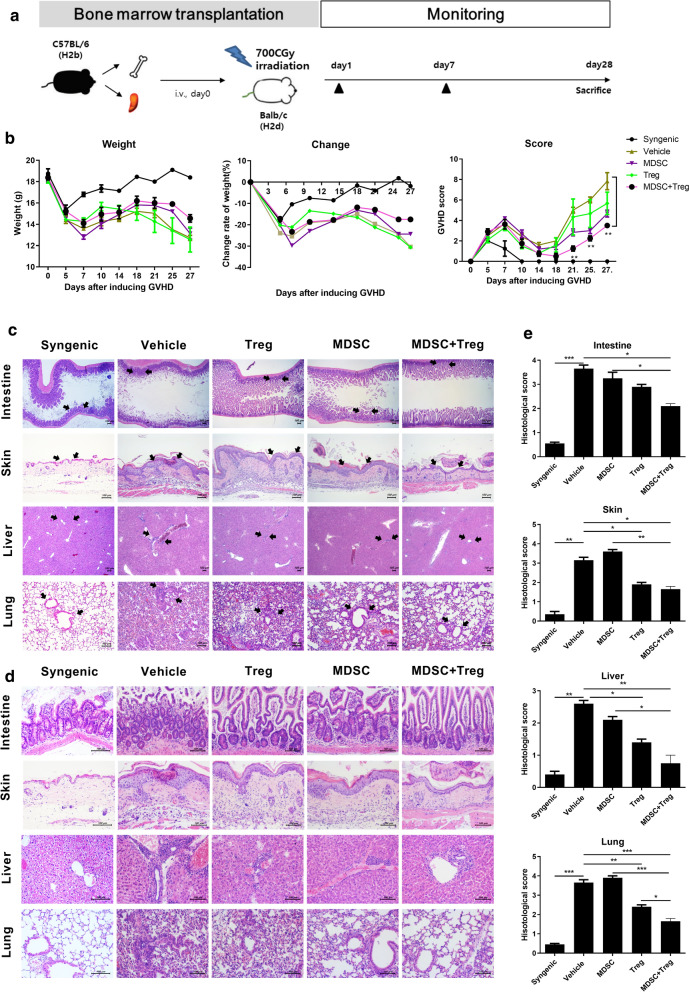
Combined cell-therapy with MDSCs and Treg attenuate the severity of aGVHD.** a** Schematic representation of the development of acute GVHD after allogeneic BMT. Splenocytes (5 × 10^6^) plus bone marrow cells (5 × 10^6^) from Balb/c (syngeneic, n = 3) or B6 mice (allogeneic, n = 15) were transplanted into irradiated B/c mice (recipient, n = 25) via intra-vein injection. After BMT, recipients were divided into 5 groups (n = 10 per group) followed by IV injection with MDSCs (1 × 10^6^) and Tregs (1 × 10^6^) individually or in combination (MDSC + Treg, MT) on days 1, 7. **b** Weight, weight change, clinical score were monitored in mice with aGVHD. Combined data from 2 independent experiments are displayed. **c** Histopathological analysis of the small intestine, skin, liver and lung at 28 days after BMT. The sections were stained with hematoxylin and eosin (Intestine, liver:original magnification, × 40, skin, lung:original magnification, × 100). **d** The lower panel (original magnification, × 200) is the zoom-in image in the arrow region of the left panel. **e** Bar represent the average the histological scores of the small intestine, skin, liver and lung. Data are representative of 2 independent experiments (*p < 0.05, **p < 0.01, ***p < 0.001 versus control (vehicle) GVHD)

To evaluate whether combined cell-therapy with MDSCs and Treg could therapeutic potential for aGVHD model. We used a fully MHC-mismatched (BALB/c (H-2k^d^) → C57BL/6 (H-2 kb) murine models (Fig. 3a). There were no differences in weight loss among the groups, while the animals treated with the combination had lower clinical scores than the control and MSC- or Treg-alone treated animals (Fig. 3b). The skin, liver, lung, and intestine are the primary targets of aGVHD. According to histopathologic analyses of the intestine, skin, liver and lung, the extent of tissue damage, inflammation, and lymphocyte infiltration was significantly reduced by the combined cell therapy compared to those of control mice and MDSCs or Tregs alone treated mice (Fig. 3c–e).

**Fig. 4:**
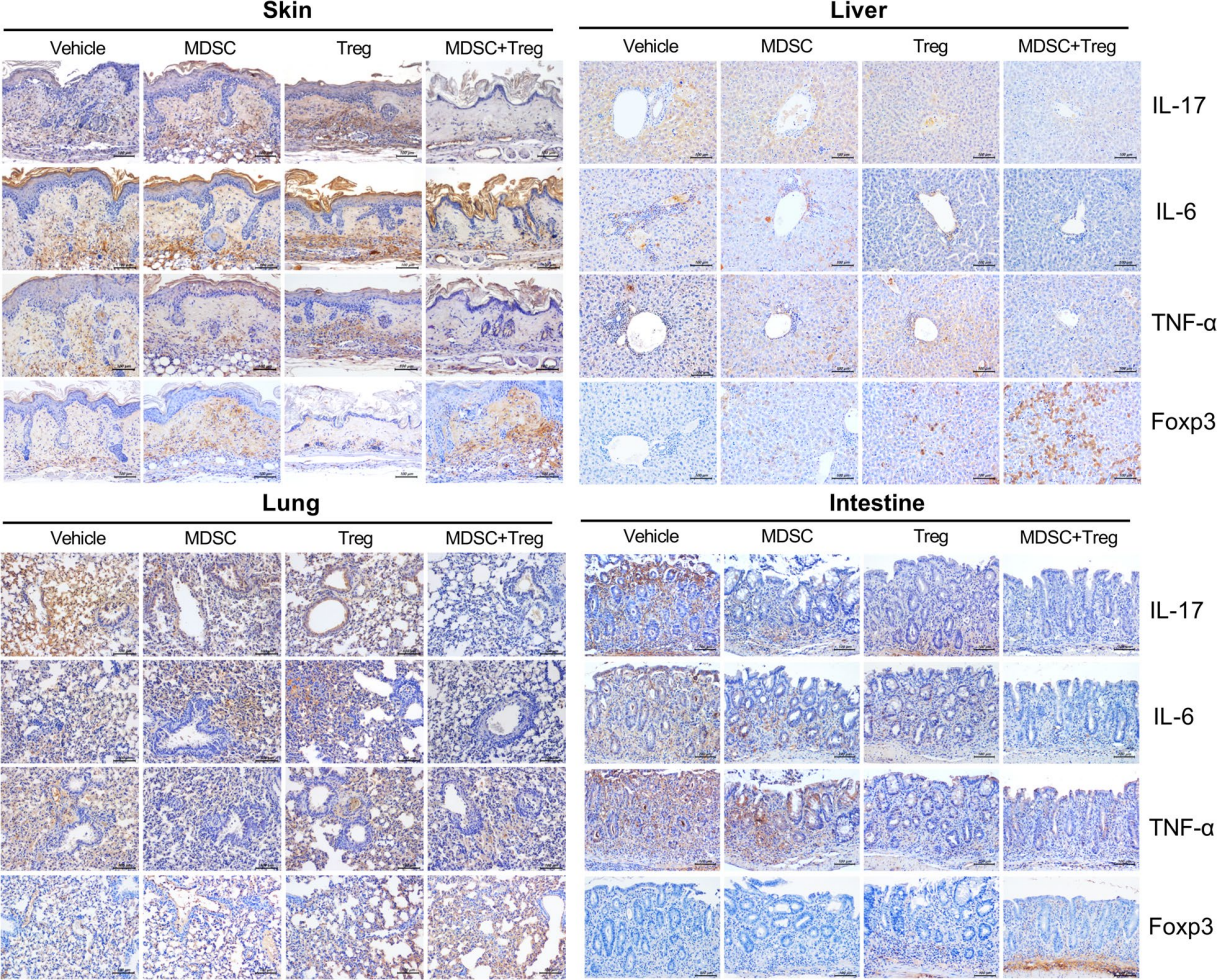
Combined cell-therapy with MDSCs and Treg decreases inflammatory cytokine expression. Immunohistochemical staining was performed to measure the expression of interleukin (IL)-17, IL-6, tumor necrosis factor (TNF)-α and Foxp3 in skin, liver, lung, intestine tissue from each groups at 28 days after BMT. (scale bar, 100 μM)

Proinflammatory cytokines, including IL-6, IL-17 and TNF-α, are key mediators of injury to target organs. To determine whether combined therapy with MDSCs and Tregs modulate the production of proinflammatory cytokines in target tissues, we performed immunohistological staining of IL-17, IL-6, TNF-α, and Foxp3 in the skin, liver, lung, and small intestine. As shown in Fig. 4 and Additional file 3: Figure S2, the expression level of IL-17, IL-6, and TNF-α was lower in sections of skin, liver, lung, and intestine tissue by the combined cell therapy compared to the control and to each cell type individually. Foxp3 + cells were also observed in the stroma region of target tissue, but majority of Foxp3 + cells was observed in lymphocyte infiltration region. Combined treatment significantly increased Foxp3 + cells compared with single treatment (Additional file 3: Figure S2).

### The combination of MDSCs and Tregs modulates T- and B-cell populations in vivo

**Fig. 5:**
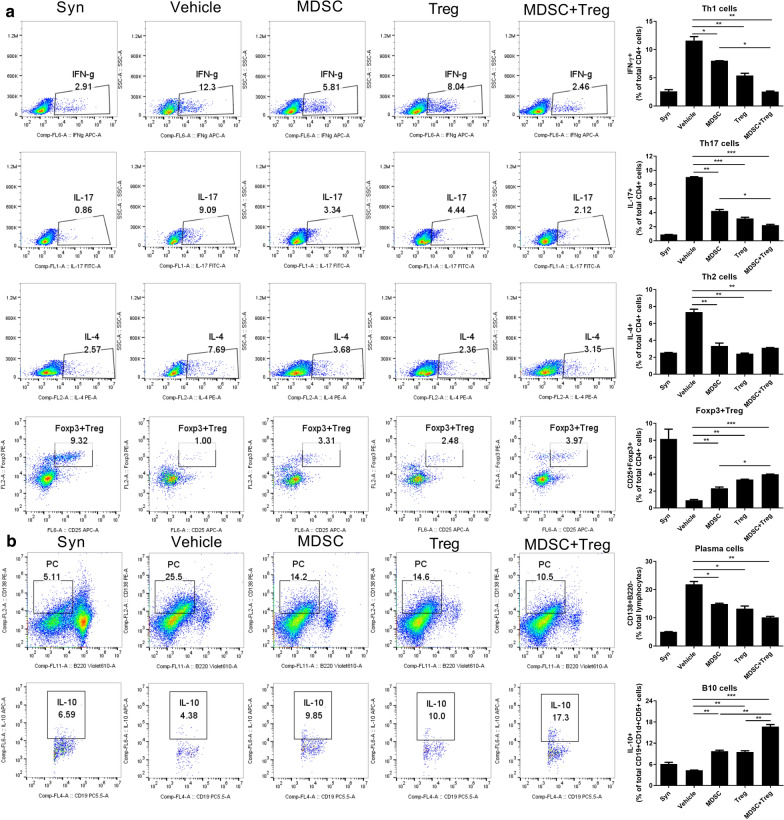
Combined cell-therapy with MDSCs and Treg altered the subpopulation of T and B cells in mice with aGVHD.** a** Splenocytes (5 × 10^6^) plus bone marrow cells (5 × 10^6^) from B6 mice were transplanted into irradiated B/c mice via intra-vein injection. Twenty-eight days after BMT, splenocytes from each group of mice were isolated and the expression levels of IL-4, IL-17, IFN-γ, and Foxp3 were determined by flow cytometry. This experiment was performed once using 6 mice. **b** Twenty-eight days after BMT, splenocytes isolated from each group of mice stained with antibodies against distinguishing B cell subsets. The percentage of B220^−^CD138^+^ plasma cells and CD1d^+^CD5^+^IL-10 producing B cells were analyzed by flow cytometry. Representative data from 2 independent experiments are shown *p < 0.05; **p < 0.01; ***p < 0.001). Numbers in the plots indicate percentages of gated cells

To determine the in vivo mechanism of action of the combination cell therapy during the development of aGVHD, we analyzed the T helper subsets by flow cytometry. In peripheral blood, the Th1 and Th17 cells were significantily reduced by the combination of MDSCs and Tregs (Additional file 4: Figure S3). The population of Th2 cells slightly expanded and did not differ among the groups. Interestingly, the Foxp3 + Treg population was increased by the combined cell therapy compared to the control and to treatment with MDSCs or Tregs alone (Additional file 1: Figure S1). Furthermore, the frequency of Th1 and Th17 cell in the spleen were significantly decreased by the combination therapy with MDSCs and the populations of Tregs. In contrast, the proportion of Foxp3 + Tregs (Fig. 5a) and IL-10 + Bregs were markedly increased (Fig. 5b).

To explore the regulatory effects of the combined cell treatment on B-cell subsets in vivo, we analyzed the populations of germinal-center B cells, plasma cells, and IL-10 producing Bregs by flow cytometry. The combination significantly decreased the populations of germinal-center B cells and plasma cells. Interestingly, the population of IL-10 + Bregs was increased by the combined cell therapy compared to the control and treatment with MDSCs or Tregs alone (Fig. 5b).

### Effects of combined therapy with MDSCs and Tregs on human T- and B-cell subsets

**Fig. 6:**
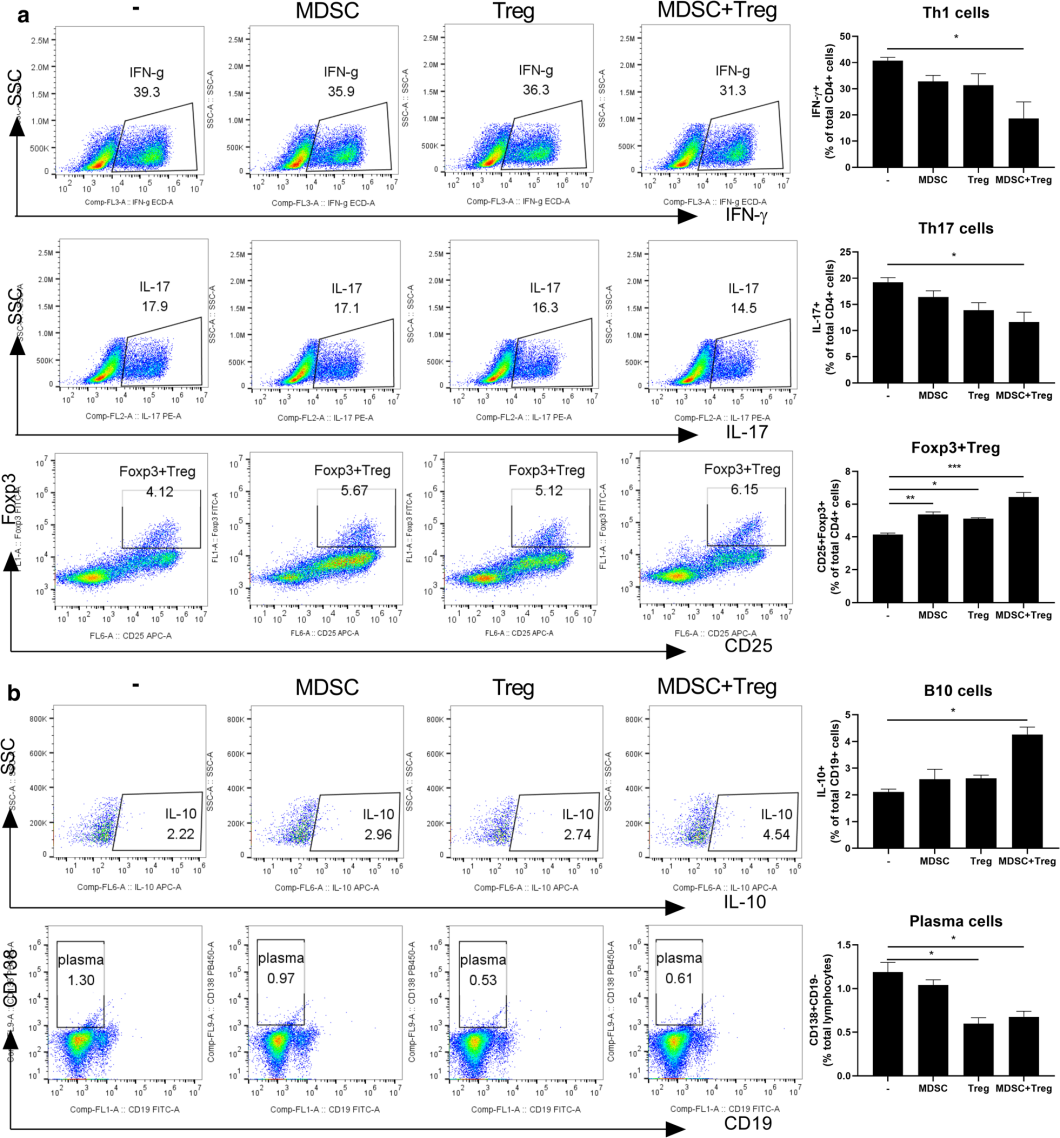
Combined treatment with MDSCs and Treg regulates human T cell and B cell response.** a** CD4 + T cells from healthy donor PBMC were coculture with MDSC or Treg alone or combined MDSC and Treg cells for 3 days and analyzed by flow cytometry. A plot from one representative experiment displays the proportions of IL-17 + , IFN-γ + , CD25 + Foxp3 + cells among CD4 + T cells. Numbers in the plots indicate percentages of gated cells. **b** Total splenocytes of normal C57BL/6 mice coculture with MDSC or Treg alone or combined MDSC and Treg cells for 3 days and analyzed by flow cytometry. A plot from one representative experiment displays the proportions of IL-10 + CD19 + cells, CD138 + B220- cells. Numbers in the plots indicate percentages of gated cells. Data are means ± SEMs. Data are representative of three independent experiments (*p < 0.05, **p < 0.01). Numbers in the plots indicate percentages of gated cells

To determine the effects of combined therapy on human T-cell subsets, we analyzed the populations of effector T cell subsets by flow cytometry. As shown in Fig. 6a, in vitro coculture with MDSCs and Tregs reduced the populations of Th1 and Th17 cells compared to coculture with either of those cell types individually. By contrast, the population of Foxp3 + Tregs was increased by the combined cell therapy compared to the control and treatment with MDSCs or Tregs alone.

Furthermore, the proportion of IL-10-producing B cells was increased by the combination cell therapy compared to the control and treatment with MDSCs or Tregs alone (Fig. 6b). However, the population of CD138 + plasma cells did not differ among the groups.

## Discussion

GVHD may develop after allogeneic HSCT is used to treat hematologic malignancies. The standard therapy for aGVHD is steroids and calcineurin inhibitors, but the long-term use of such nonspecific immunosuppressive drugs causes severe side effects [5]. Thus, more effective treatment strategies for acute GVHD urgently needed.

MDSCs are potent immunosuppressive cells in various pathologic settings. They can inhibit T-cell responses both in vitro and in vivo by producing factors such as arginase 1, iNOS, reactive oxygen species, and TGF-β, which inactivate a variety of immune cell types, particularly T cells [6]. MDSC induce expansion of Tregs [28, 33]. In addition, MDSCs promote the expansion of Breg cells (IL-10-producing B cells) via an iNOS-dependent pathway and ameliorate autoimmune disorders [14].

In recent studies, MDSCs have potential for the treatment of GVHD [16, 17, 34]. However, our data show that MDSCs alone had low clinical efficacy. Highfill et al. showed that an IL-13-producing subset of MDSCs had a greater suppressive effect than MDSCs [15]. MDSC show promise as potential cell therapy for the treatment GVHD, however the regulatory role of myeloid cells is poorly understood and still lack of a clinical research for the application.

Tregs can control GVHD in mice [35, 36] and humans [37, 38] by inhibiting the alloreactive T-cell response. They have shown positive results in preclinical trials and their ability to ameliorate GVHD is under investigation. However, this approach requires standardization of Treg expansion methods and doses [39, 40]. Inducible Tregs can readily be generated, but controversial preclinical findings and phenotype instability have hampered their translation to the clinic [18].

In previous study, for the first time that murine MDSCs induced expansion Breg cells (IL‐10–producing B cells) have therapeutic effect in an animal model of SLE [14]. Bregs have significant immunosuppressive abilities both in vitro and in vivo. Bregs prevent the onset of GVHD by inhibiting the differentiation of Th1 and Th17 cells and promoting the expansion of Tregs [29, 41, 42]. Therefore, MDSC-mediated expansion of Bregs is important for the treatment of aGVHD. Our data confirm that our combination therapy with MDSCs Tregs has potential for ameliorating GVHD.

We first analyzed the regulatory effect of T and B cell subset by combination therapy of MDSC and Treg. As reported in previous studies, our results confirmed an increase in Treg and a decrease in Th1 and Th17 cells by MDSC or Treg cells alone, but the combination treatment confirmed a more synergistic immunomodulatory effect. The combination of MDSCs and Tregs simultaneously induced Treg skewing and Th1/Th17 suppression, increased the frequency of IL-10-producing Bregs, and decreased the frequency of plasma cells. It also controlled the proliferation of alloreactive T cells and the Treg/Th17 balance in vitro. These data suggest that the combination of MDSCs and Tregs has potent immunoregulatory effects in mice and humans.

Systemic combined treatment of MDSCs and Treg cells improved clinical GVHD severity, effectively modulating the T helper cell response, reduced the percentage of alloreactive of Th1, and Th17 cells, and increased the frequencies of Th2 and Foxp3 + Treg cells. In vivo, the combination decreased the population of germinal center B cells and plasma cells and increased that of IL-10-producing Bregs. Thus, the combination cell therapy directly modulated the reciprocal regulation of CD4 + T-cell and B-cell subsets. Moreover, the expression of proinflammatory cytokine (IL-6, IL-17 and TNF-α) decreased, and that of Foxp3 increased, in the GVHD target tissues (skin, liver, lung, and intestine) from mice treated with MDSC and Treg, suggesting that the in vivo suppressive activity of the combination therapy is attributable in part to soluble mediators. Future studies should investigate the immunoregulatory mechanisms and the interactions of the combination of MDSCs and Tregs for their therapeutic application.

Previous reports suggest that the combination of MSCs and Treg cell therapy have enhanced therapeutic efficacy in transplantation model [43–45]. However, the mechanism underlying cross talk between MSC and Treg remains unclear [46]. MSC therapy have also been proposed as a treatment for steroid-refractory GVHD but the results are still controversial [47, 48]. Furthermore the origin, delivery route, dose, and timing of infused MSCs influences therapeutic efficacy. MSC therapy is also insufficient to inhibit production of pro-inflammatory cytokines and reversed MSC-mediated therapetic efficacy under inflammatory condition [49, 50]. It was reported that procoagulant tissue factor expression of MSCs by intravascular infusion trigger the instant blood-mediated inflammatory reaction (IBMIR), which in turn activate coagulation. These various problems in MSC therapy impede clinical applications [51]. We therefore suggest that MDSCs plus Treg combination therapy would be an alternative new treatment against GVHD that overcomes the limitations of MSC based therapy. Human MDSC therapy has been difficult to use clinically due to the limited number of MDSCs. However, recent studies have shown that the development of large-scale extended MDSCs offers the potential for use in clinical research. [17]

Our research has some limitations. First, only female recipient and donor mice were used. Minor histocompatibility associated with sex chromosomes affects the incidence and severity of the GHVD model. Gender-related factors have a significant impact on HSCT results [52, 53]. Our model requires further research to confirm the therapeutic effects of sex differentiation between recipients and donors. Second, the combination of MDSC and Treg did not demonstrate a GVL effect. To optimize the balance between GVHD and GVL in allogeneic stem cell transplantation, allogeneic HSCT must be successful. MDSC improved the severity and mortality of GVHD while maintaining GVL activity [54]. Allogeneic iTregs can weaken GVHD, but can significantly impair GVL function [55]. Further research is needed to confirm the GVL effect of the combination of MDSC and Treg. Despite these limitations, this was the first study to evaluate the effectiveness of MDSC and Treg combination therapy in a GVHD model. Previous studies reported an inhibitory effect on GVHD with a single treatment of each MDSC and Treg, but this study showed a synergistic effect of GVHD treatment with a combination of MDSC and Treg. Therefore, the results of this study provide a potential strategy for mitigating GVHD by overcoming the limitations of future single-cell therapies.

## Conclusion

In conclusion, the combination of MDSCs and Tregs had a synergistic immunoregulatory effect by increasing the Treg/Breg populations and decreasing the populations of effector Th1 and Th17 cells, which ameliorated GVHD development. Taken together, this study demonstrates that combined therapy of MDSCs and Treg cells with fewer side effects than current immunosuppressant drugs has potential for treating aGVHD and should be evaluated in clinical trials.

## Supplementary Information

**Additional file 1: Table S1.** Clinical GVHD scoring system.

**Additional file 2: Figure S1.** In vitro does test of combined cell-therapy with MDSC and Treg on Treg/Th17 regulation.

**Additional file 3: Figure S2.** Combined cell-therapy with MDSCs and Treg decreases inflammatory cytokine and Foxp3 expression.

**Additional file 4: Figure S3.** Combined cell-therapy with MDSCs and Treg altered the subpopulation of T cell in peripheral blood.

## Data Availability

All data are available in the manuscript or upon request to the authors.

## Abbreviations

MDSC: Myeloid-derived suppressor cells
Tregs: Regulatory T cells
GHVD: Graft-versus-host disease
HSCT: Hematopoietic stem cell transplantation
BMT: Bone marrow transplantation
BMC: Bone marrow-derived cells
APCs: Antigen-presenting cells
iNOS: Inducible nitric oxide synthetase
SLE: Systemic lupus erythematosus
IBD: Inflammatory bowel disease
IFN-γ: Interferon-γ
IL: Interleukin
Th: T helper
