# Supplementary material for: Myeloid-derived suppressor cells therapy enhance immunoregulatory properties in acute graft versus host disease with combination of regulatory T cells
Source: J Transl Med. 2020 Dec 14;18:483. doi: 10.1186/s12967-020-02657-6 (PMC7734831; doi:10.1186/s12967-020-02657-6)
Supplement: Supplementary file 1 — Additional file 1: Table S1. Clinical GVHD scoring system. [file 12967_2020_2657_MOESM1_ESM.pdf]

Table S1. Clinical GVHD scoring system

Criteria	Grade 0	Grade 1	Grade 2
Weight loss	<10%	>10% to <25%	>25%
Posture	Normal	Hunching at rest	Severe hunching, impaired movement
Activity	Normal	Mild to moderate decrease	Severe decrease
Fur texture	Normal	Mild to moderate ruffling	Severe ruffling, poor grooming
Skin Integrity	Normal	Scaling of paws and/or tail	Obvious areas of denuded skin
