# Supplementary material for: Myeloid-derived suppressor cells therapy enhance immunoregulatory properties in acute graft versus host disease with combination of regulatory T cells
Source: J Transl Med. 2020 Dec 14;18:483. doi: 10.1186/s12967-020-02657-6 (PMC7734831; doi:10.1186/s12967-020-02657-6)
Supplement: Supplementary file 2 — Additional file 2: Figure S1. In vitro does test of combined cell-therapy with MDSC and Treg on Treg/Th17 regulation. [file 12967_2020_2657_MOESM2_ESM.pdf]

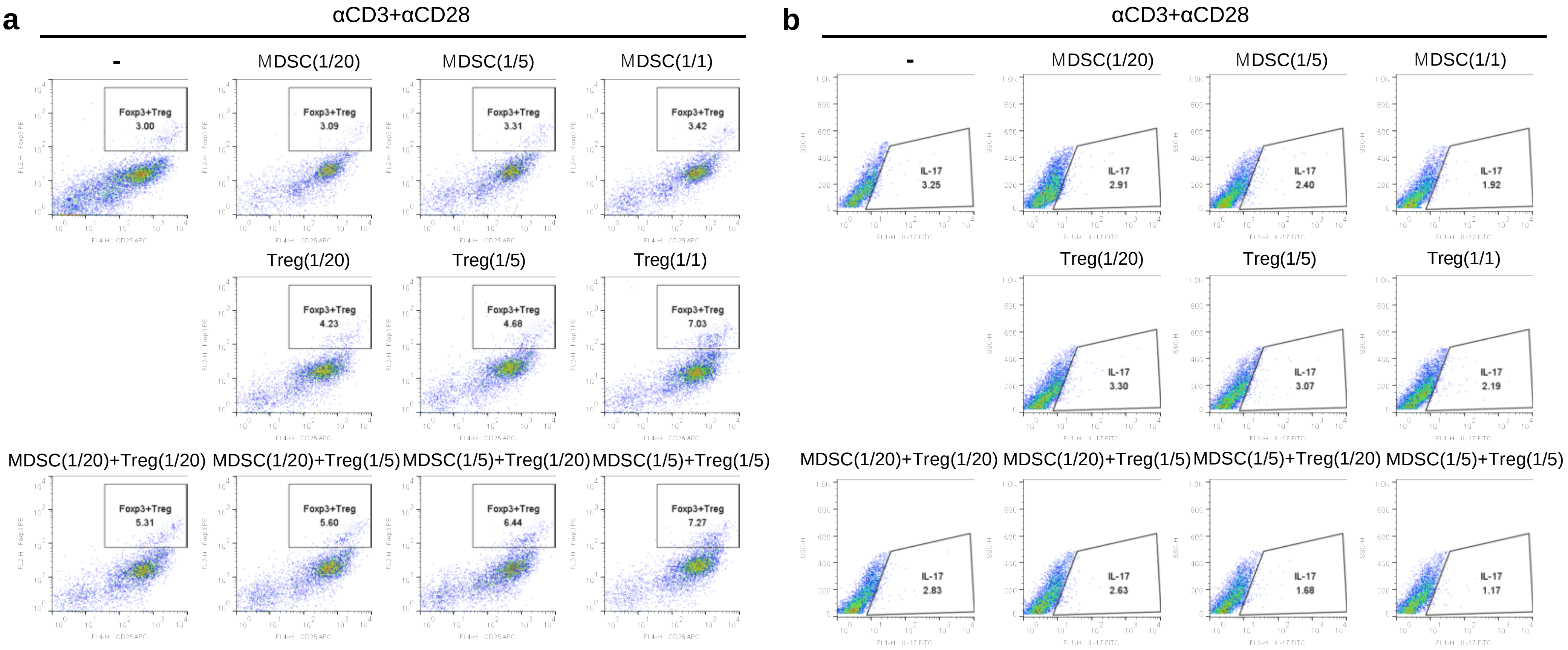

Figure S1. in vitro does test of combined cell-therapy with MDSC and Treg on Treg/Th17 regulation

CD4+T cell(1×10^6) isolated from normal C57BL/6 mice were cocultured with MDSC or Treg alone or combined MDSC and Treg cells for 3 days in the presence of anti-mouse CD3 and CD28 antibody and analyzed by flow cytometry. A plot from one representative experiment displays the proportions of IL-17+, CD25+Foxp3+ cells among CD4+ T cells. Numbers in the plots indicate percentages of gated cells.
