# Supplementary material for: Myeloid-derived suppressor cells therapy enhance immunoregulatory properties in acute graft versus host disease with combination of regulatory T cells
Source: J Transl Med. 2020 Dec 14;18:483. doi: 10.1186/s12967-020-02657-6 (PMC7734831; doi:10.1186/s12967-020-02657-6)
Supplement: Supplementary file 3 — Additional file 3: Figure S2. Combined cell-therapy with MDSCs and Treg decreases inflammatory cytokine and Foxp3 expression. [file 12967_2020_2657_MOESM3_ESM.pdf]

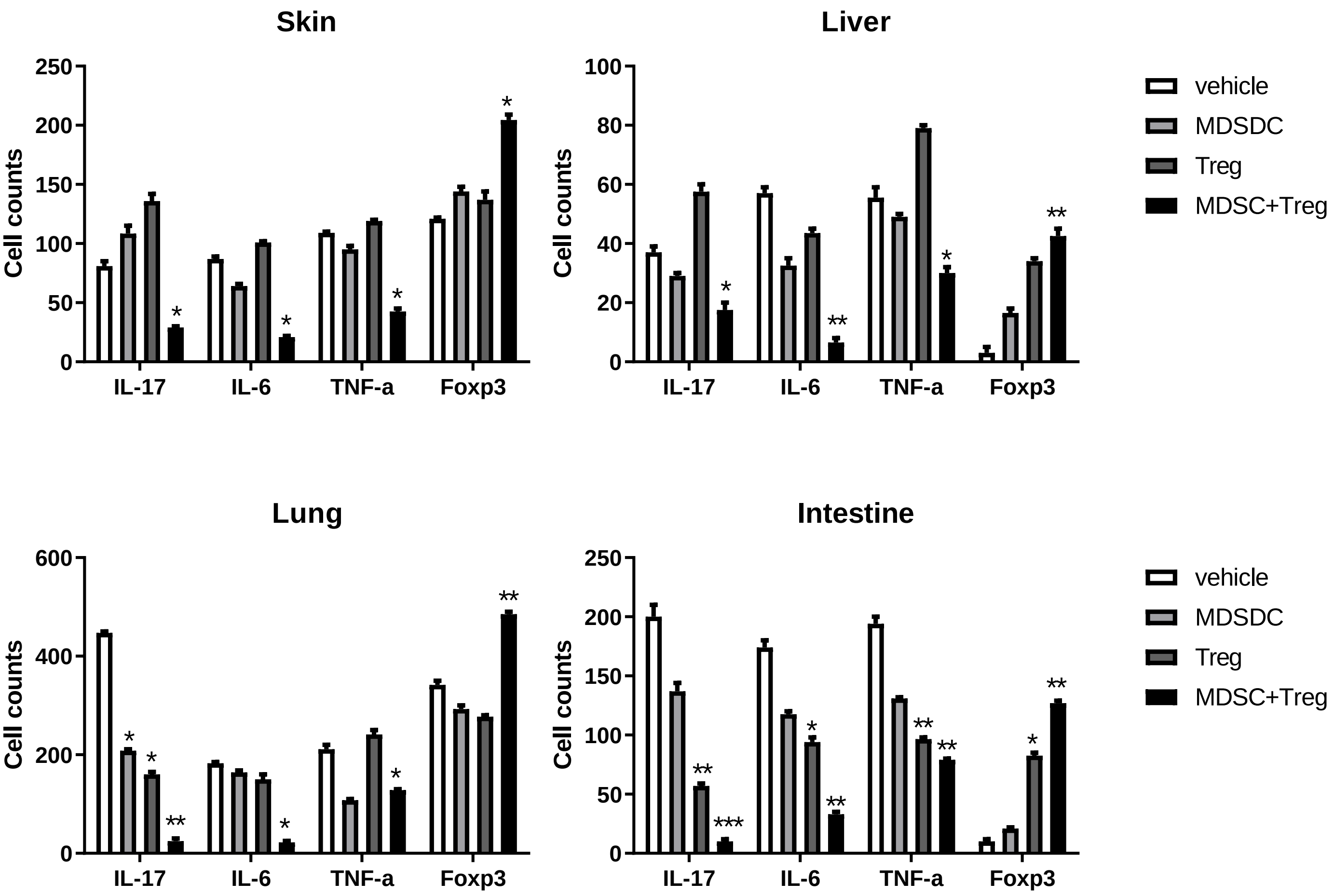

Figure S2. Combined cell-therapy with MDSCs and Treg decreases inflammatory cytokine and Foxp3 expression.

Immunohistochemical staining was performed to measure the expression of interleukin (IL)-17, IL-6, tumor necrosis factor (TNF)-α and Foxp3 in skin, liver, lung, intestine tissue from each groups at 28 days after BMT. (scale bar, 100 μM). The positive cells for each antibody are shown. Data reprsent the mean ± SEM of 3 independent experiments. *$p < 0.05$; **$p < 0.01$; ***$p < 0.001$ (bars indicate the means).
