# Supplementary figures and images for: Myeloid-derived suppressor cells therapy enhance immunoregulatory properties in acute graft versus host disease with combination of regulatory T cells
Source: J Transl Med. 2020 Dec 14;18:483. doi: 10.1186/s12967-020-02657-6 (PMC7734831; doi:10.1186/s12967-020-02657-6)

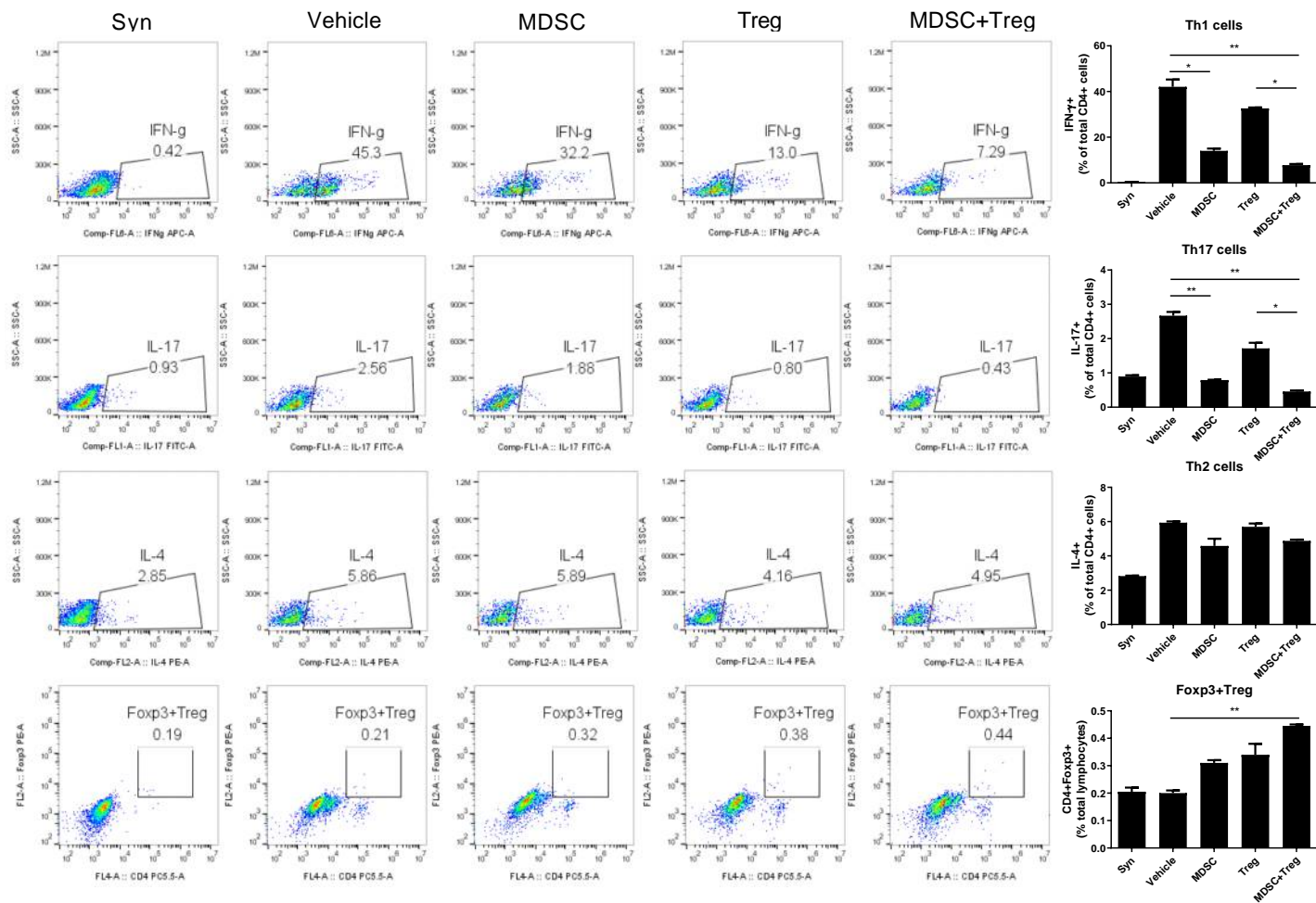

Supplement: Supplementary file 4 — Additional file 4: Figure S3. Combined cell-therapy with MDSCs and Treg altered the subpopulation of T cell in peripheral blood. [file 12967_2020_2657_MOESM4_ESM.pdf]
